# The Safety and Effectiveness of Laparoscopic Pyloromyotomy Using 3-mm Electrocautery Hook versus Open Surgery for Treatment of Hypertrophic Pyloric Stenosis in Infants

**DOI:** 10.3390/children8080701

**Published:** 2021-08-13

**Authors:** Zenon Pogorelić, Ana Zelić, Miro Jukić, Carlos Martin Llorente Muñoz

**Affiliations:** 1 Department of Pediatric Surgery, University Hospital of Split, 21 000 Split, Croatia; mirojukic.mefst@gmail.com; 2 Department of Surgery, School of Medicine, University of Split, 21 000 Split, Croatia; anazelic@hotmail.com; 3 Surgical Clinic Medix-Muñoz, 28 000 Madrid, Spain; llorentecm@gmail.com

**Keywords:** hypertrophic pyloric stenosis, pyloromyotomy, laparoscopic pyloromyotomy, open pyloromyotomy, electrocautery hook, outcomes of treatment, infants

## Abstract

Background: The standard of treatment for infants with hypertrophic pyloric stenosis is still pyloromyotomy. Recently, in most of the pediatric surgery centers laparoscopic pyloromyotomy has become popular. The aim of the present study is to compare the outcomes of treatment in infants with hypertrophic pyloric stenosis between traditional open approach and laparoscopic pyloromyotomy using 3-mm electrocautery hook. Methods: A total of 125 infants, 104 (83.2%) males, with median age 33 (interquartile range, IQR 24, 40) days, who underwent pyloromyotomy because of hypertrophic pyloric stenosis, between 2005 and 2021, were included in the retrospective study. Of that number 61 (48.8%) infants were allocated to the open group and 64 (51.2%) to the laparoscopic group. The groups were compared in regards to time to oral intake, duration of surgery, the type and rate of complications, rate of reoperations, frequency of vomiting after surgery, and the length of hospital stay. Results: No differences were found with regards to baseline characteristics between two investigated groups. Laparoscopic approach was associated with significantly better outcomes compared to open approach: shorter duration of surgery (35 min (IQR 30, 45) vs. 45 min (40, 57.5); *p* = 0.00008), shorter time to oral intake (6 h (IQR 4, 8) vs. 22 h (13.5, 24); *p* < 0.00001), lower frequency of postoperative vomiting (*n* = 10 (15.6%) vs. *n* = 19 (31.1%)), and shorter length of postoperative hospital stay (3 days (IQR 2, 3) vs. 6 days (4.5, 8); *p* < 0.00001). In regards to complications and reoperation rates, both were lower in the laparoscopic pyloromyotomy group but the differences were not statistically significant (*p* = 0.157 and *p* = 0.113, respectively). The most common complication in both groups was mucosal perforation (open group, *n* = 3 (4.9%); laparoscopic group, *n* = 2 (3.1%)) followed by wound infection in open group, *n* = 3 (4.9%). No cases of wound infection were recorded in the laparoscopic group. Conclusion: Open and laparoscopic pyloromyotomy are equally safe and effective in treatment of hypertrophic pyloric stenosis. Laparoscopic technique is associated with faster recovery, shorter duration of surgery and shorter duration of hospital stay.

## 1. Introduction

Hypertrophic pyloric stenosis is the most common surgical disorder of the stomach in infants producing non-bilious vomiting. Circular and longitudinal muscular fibers of the pylorus and the distal antrum of the stomach gradually hypertrophied causing thickening of the pylorus and progressive narrowing of the pyloric canal [1]. Exact cause of this condition is not known. Until now several possible causes and factors have been suggested such as antibiotics (macrolydes, eritromycin), cesarean section, bottle feeding, preterm delivery, first born infants, and smoking during the pregnancy, but none of them have been proven [1,2,3]. An incidence of hypertrophic pyloric stenosis is 2–5 infants per 1000 live births yearly. It is four times more common in male than female newborns. It is most commonly seen in white population, while it is rare in Black and Asian populations [1,4]. 

At birth newborns are well and usually tolerate meals properly for the first two weeks. This condition, in most of the cases, occurs between the third and sixth week of life [1,4]. In very rare cases late onset presentation of hypertrophic pyloric stenosis has been described, with age ranging from three months to five years of age [5]. In the beginning, infants occasionally vomit after the meal. However, as the disease progresses the infants present with typical clinical appearance of hypertrophic pyloric stenosis, projectile vomiting after every meal, which if left untreated may potentially lead to dehydration, electrolyte imbalance, and weight loss [1,2,3,4,6]. In a certain percentage of infants, a hard, non-tender pylorus (so-called olive) may be palpated near the epigastrium. Rarely, reverse peristaltic waves of the stomach may be visible. 

Due to frequent vomiting typical metabolic imbalance of hypertrophic pyloric stenosis causes metabolic alkalosis (body’s pH < 7.45), which in late presented cases or in cases with severe vomiting is often associated with hypochloremia (serum chloride level <96 mEq/L) and hypokalemia (serum potassium level <3.5 mEq/L) [7,8]. Previously barium enema with upper gastrointestinal series was the standard diagnostic method for diagnosing hypertrophic pyloric stenosis. Nowadays, abdominal ultrasound has become the standard diagnostic tool method [9]. The ultrasound criteria for the hypertrophic pyloric stenosis include muscle wall thickness >4 mm, length of pyloric canal >16 mm, and diameter of the pylorus >12 mm [9,10]. Initial treatment is placement of a nasogastric tube, cessation of feeding and rehydration, and correction of electrolyte and metabolic imbalances [11]. 

Although there are reports in literature of treatment of hypertrophic pyloric stenosis with atropine-sulfate, in most centers the standard approach to treatment of this condition is surgery, i.e., pyloromyotomy. The success rates of conservative treatment with atropine-sulfate varies between 68 and 87% [11,12,13,14]. The surgical approach involves pyloromyotomy, which can be performed in the traditional open way or, more recently, by laparoscopic approach [6,11]. The traditional open technique is well-known with adequate postoperative effects but recovery after surgery lasts a little longer due to slower oral intake and a higher level of pain due to abdominal incision, cosmetic effects are poor as well [6,11,15].

Recently, with breakthrough of laparoscopic surgery among the pediatric surgeons laparoscopic pyloromyotomy has been more frequently used due to its benefits, such as significantly lesser duration of surgery, faster oral intake, faster recovery, lesser pain and tissue trauma, and excellent cosmetic effects [6,11,15,16,17,18]. In regards to efficacy of the procedure a meta-analysis confirmed that laparoscopic approach had the same safety and efficacy as open surgery [15]. 

In the majority of the centers traditional laparoscopic pyloromyotomy is performed using a retractable pyloromyotomy knife for incision of the hypertrophied pylorus [5,6,15,16,17,18]. However, in all centers, especially in countries with lower socio-economic status, such an instrument is not always available, so pediatric surgeons used various modifications of this knife to perform the procedure by laparoscopic approach. Shah et al. used a tip of surgical blade which was placed in a laparoscopic needle holder [19]. The same technique was confirmed in a recent study by Ramji et al. from Africa [20]. Such an alternative is not without danger since the knife from the needle holder can fall out into the abdominal cavity and possibly cause injury to surrounding structures or organs. Moreover, when inserting a needle holder with a knife in it can cause injuries to the abdominal organs. Hypothetically, the tip of the knife if dropped out of needle holder may sometimes be difficult to identify by a laparoscopic approach which can further complicate the procedure and be a reason for laparotomy [19,20]. Abu-Kishk described a myringotomy knife as an alternative for a retractable pyloromyotomy knife [21]. Some pediatric surgeons found an alternative in a single-use disposable arthrotomy knife but that knife is no longer available [22]. Moreover, the use of a retractable pyloromyotomy knife in some cases may be associated with bleeding from pyloric muscle. By cutting the pyloric muscle, some of the smaller blood vessels on its surface can be opened, which can be the source of bleeding and contamination of the visual field with blood [22].

Most recently use of a hook with electrocautery was reported instead of the use of a retractable pyloromyotomy knife with excellent outcomes [22]. Jain et al. first reported usage of an electrocautery hook in a cohort of 15 patients, as a valuable alternative to a retractable pyloromyotomy knife. They concluded that pyloromyotomy using an electrocautery hook is as safe and effective as the usage of a retractable pyloromyotomy knife. Moreover, usage of electrocautery hook results in a bloodless operative field, which is very important to pediatric laparoscopic surgeons who work in very limited space in infants [22]. The use of electrocautery hook may also lead to complications such as thermal damage and opening of the pyloric mucosa but as the 3-mm hook 3 is always used in infants with hypertrophic pyloric stenosis the risk of mucosal injury is very low due to the fact that the thickness of the hypertrophic pyloric muscle in most of the cases is greater than 4 mm. The hook could therefore be inserted liberally in the pylorus to divide the muscle at depth.

To the best of our knowledge only one study (in a limited number of patients, *n* = 15) reported successful usage of electrocautery hook in pediatric patients with hypertrophic pyloric stenosis. The aim of the present study was to compare outcomes of treatment in infants with hypertrophic pyloric stenosis between traditional open approach and laparoscopic pyloromyotomy using 3-mm electrocautery hook in a larger cohort of patients to confirm its value in everyday practice.

## 2. Materials and Methods

### 2.1. Patients

The case records of 132 infants with median age of 35 (interquartile range, IQR 24, 40) days, who underwent surgery because of hypertrophic pyloric stenosis, in a period from 1 January 2005 to 1 July 2021, were retrospectively reviewed. Out of 132 identified infants, 7 were excluded because they met one or more of the exclusion criteria, so finally 125 patients were included for analysis. Inclusion criteria was infants of both sexes who underwent surgery because of hypertrophic pyloric stenosis. Exclusion criteria were: negative intraoperative finding of hypertrophic pyloric stenosis, pyloric stenosis of other etiology, pyloric stenosis outside the neonatal or infant age, conservative treatment of hypertrophic pyloric stenosis, and incomplete data in case records of the patient.

### 2.2. Outcomes of the Study

The primary outcome of the study was a comparison of treatment outcomes between the two study groups, which included the time to establishment of intestinal peristalsis, time to oral intake and the type, duration of surgery, and rate of complications. Secondary outcome measures were the rate of vomiting after surgery and the length of hospital stay.

### 2.3. Study Design

The following parameters were analyzed for each patient who has been diagnosed with hypertrophic pyloric stenosis and who has undergone open or laparoscopic surgery: age, gender, duration of symptoms, body weight, laboratory parameters (presence of metabolic alkalosis, hypochloremia, and hypokalemia). Ultrasound findings (muscle wall thickness, length, and diameter of pyloric canal), presence of associated anomalies, duration of surgery, time to oral intake, use of analgesia, postoperative vomiting, length of hospital stay, and complications. These parameters were compared between the two examined groups and with data from the available medical literature. For the purpose of this study the patients were divided into two study groups, depending on surgical approach. The patients from the first group received traditional open pyloromyotomy while the patients from the second group underwent laparoscopic pyloromyotomy. The technique used for pyloromyotomy was chosen by the operating surgeon. The study was approved by the Ethics Review Board of our hospital (reference No. 500-03/21-01/116).

### 2.4. Surgical Technique

#### 2.4.1. Open Pyloromyotomy

Abdominal cavity is opened through the right upper quadrant transverse skin incision. After mobilization of the stomach the hypertrophied pylorus is exposed through the abdominal incision. Pyloromyotomy is performed from the antrum of the stomach to the prepyloric vein of Mayo. The muscle edges of hypertrophied pylorus are dissected using a blunt instrument. The procedure is terminated when the mucosa protrudes through the incision. After securing hemostasis abdominal wall is closed in layers.

#### 2.4.2. Laparoscopic Pyloromyotomy

**Figure 1:**
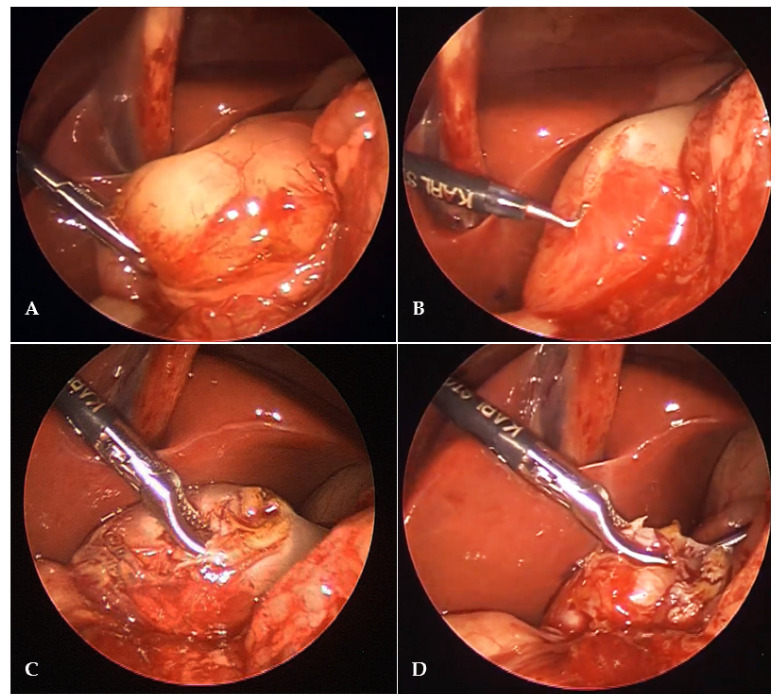
Intraoperative finding in 14-day-old male infant with hypertrophic pyloric stenosis who received laparoscopic pyloromyotomy: (**A**) Laparoscopic view of hypertrophied pylorus; (**B**) pyloromiotomy using 3-mm diathermy hook; (**C**) separation of the pyloric muscle layer by blunt dissection; (**D**) end of the procedure, mucosa clearly protrudes trough the incision.

A mini incision is performed in the supraumbilical region. Pneumoperitoneum is established through the Veress needle. A level of pneumoperitoneum is set at 6 mmHg. A 3.5-mm trocar is inserted through previous supraumbilical incision. A 3.5-mm laparoscope (Karl Storz, Tuttlingen, Germany) is used to inspect the abdominal cavity. After the hypertrophied pylorus is identified, additional two lateral 3.5-mm ports are introduced at left and right subcostal region 1–2 cm laterally to the midclavicular line. Pylorus is grasped using pyloric grasper (Karl Storz, Tuttlingen, Germany) (Figure 1A). Pyloromyotomy is performed using a 3-mm diathermy hook (Karl Storz, Tuttlingen, Germany) from the antrum of the stomach to the prepyloric vein of Mayo (Figure 1B). The muscle edges of hypertrophied pylorus are dissected using an endo-dissector (Karl Storz, Tuttlingen, Germany) (Figure 1C). The procedure is terminated when the mucosa protrudes through the incision (Figure 1D). At the end of the procedure an air insufflation through nasogastric tube into stomach for testing seromucosal integrity was performed. After the hemostasis is secured, ports are removed and skin incisions are closed using braided adhesive skin closures (3M^TM^ Steri-Strip^TM^, Neuss, Germany).

### 2.5. Postoperative Protocol and Follow-Up

After surgical procedure, the patients were observed in the intensive care unit for several hours until they were fully awake. An intravenous fluid (5% glucose + 0.45% NaCl) in a dose 4 mL/kg/h was started. Oral feeding was started with 5–10 mL of 5% glucose 2–24 h after the surgery, depending on the operating surgeon decision. If the patient tolerated glucose well, breast milk or adapted milk formula was started. The amount of milk was increased with each meal until an age-appropriate meal was achieved. In case of vomiting, the infusion was continued and after a few hours the oral milk was started again according to the same scheme in smaller meals. Paracetamol (Perfalgan, Bristol-Myers Squibb Pharmaceuticals limited, Bristol, UK) in dose of 10–15 mg/kg was used for analgesia. Afebrile patients with no vomiting within 24 h and complete tolerance of oral meals were discharged from the hospital. The children were followed-up at 7 and 30 days after discharge at outpatient clinic.

### 2.6. Statistical Analysis

Statistical analyses were conducted using SPSS 24.0 software (IBM Corp., Armonk, NY, USA). Comparative analyses were performed with Mann–Whitney U test for continuous variables and Chi-square tests for categorical variables, as appropriate. Two-sided Fisher’s exact test was used when the frequency of events in a certain cell was low. *p* values < 0.05 were considered significant.

## 3. Results

A total of 125 infants, 104 (83.2%) males, with median age 33 (IQR 24, 40) days, who received pyloromyotomy because of hypertrophic pyloric stenosis were included in the study. Of that number 61 infants were allocated to the open pyloromyotomy group and 64 infants were allocated to laparoscopic pyloromyotomy group. 

Both groups were symmetric in regards to all investigated preoperative demographic parameters: age (*p* = 0.453), gender (*p* = 0.570), weight (*p* = 0.226), malnutrition status (*p* = 0.255), incidence of associated anomalies (*p* = 1.000). There were no differences between the groups with regards to preoperative laboratory parameters: incidence of hypokalemia (*p* = 0.350), hypochloremia (*p* = 0.316), and metabolic alkalosis (*p* = 0.974). Moreover, the investigated groups did not defer in regards to preoperative ultrasound findings: pyloric wall thickness (*p* = 0.260), length of pyloric (*p* = 0.857) canal, and diameter of pylorus (*p* = 0.441). Demographic characteristics, clinical and laboratory data of patients are presented in Table 1.

Significantly shorter duration of surgery was recorded in the laparoscopic pyloromyotomy group, 35 min (IQR 30, 45) compared to the open pyloromyotomy group, 45 min (40, 57.5); *p* = 0.00008. Time to oral intake after surgery was also significantly shorter in the laparoscopic pyloromyotomy group, 6 h (IQR 4, 8) compared to the open pyloromyotomy group, 22 h (IQR 13.5, 24); *p* < 0.00001. Frequency of postoperative vomiting was significantly higher in open pyloromyotomy group, *n* = 19 (31.1%) compared to the laparoscopic pyloromyotomy group, *n* = 10 (15.6%); *p* = 0.039. Median length of postoperative hospital stay was shorter in the laparoscopic pyloromyotomy group, 3 days (IQR 2, 3) compared to the open pyloromyotomy group, 6 days (IQR 4.5, 8); *p* < 0.00001.

In regards to complications and reoperation rates, both were lower in the laparoscopic pyloromyotomy group but the differences were not statistically significant (*p* = 0.157 and *p* = 0.113, respectively). The most common complication in both groups was mucosal perforation (open group, *n* = 3 (4.9%); laparoscopic group, *n* = 2 (3.1%)) followed by wound infection in open group, *n* = 3 (4.9%). No cases of wound infections were recorded in the laparoscopic group. Three children (4.9%) from the open pyloromyotomy group required reoperation because of diffuse peritonitis caused by mucosal perforation. In the laparoscopic group both perforations were recognized intraoperatively and were secured with laparoscopic suture of the mucosa with no further consequences. Outcomes of treatment are presented in Table 2.

## 4. Discussion

In the majority of the cases of laparoscopic pyloromyotomy the pyloric cutting knife for incision of the hypertrophied pylorus has been frequently used. The results of the present study confirmed that laparoscopic pyloromyotomy using 3-mm electrocautery hook, in a hands of experienced laparoscopic surgeon, is a safe and effective method for treatment of infants with hypertrophic pyloric stenosis. This study confirmed also that surgical time, length of hospital stay, and time to oral intake after surgical procedure are significantly shorter compared to open surgery. Frequency of postoperative vomiting was also lower in children who underwent laparoscopic approach. Frequency of complications and reoperations was also lower in laparoscopic approach but without statistical significance.

Nowadays, despite several reports of conservative treatment with atropine, surgical treatment (pyloromyotomy) is still the method of choice for infants suffering from hypertrophic pyloric stenosis [11,12,13,14,23]. Traditional open pyloromyotomy was initially introduced by German surgeon Ramstedt who described the first successful cases of pyloromyotomy [24]. Since then, hypertrophic pyloric stenosis was successfully treated using a traditional open approach for many years. Last two to three decades laparoscopic surgery gained popularity among pediatric surgeons, especially after development of instruments suitable for the pediatric population. Various pediatric procedures such as hernia repair, varicocelectomy, cholecystectomy, appendectomy, pyloromyotomy etc., which until then had been performed exclusively by the open approach, began to be performed using laparoscopic approach [15,16,17,18,19,20,21,25,26,27,28,29]. Numerous studies reported benefits of laparoscopic approach for treatment of hypertrophic pyloric stenosis such as faster recovery, faster postoperative feeding, lesser pain, shorter surgical and hospitalization times and lower incidence of wound related complications [6,11,15,16,17,18,19,20,21,22]. The cosmetic results are incomparably better in infants operated using laparoscopic approach [6,11,15,16,17,18,19,20,21,22]. The success and/or superiority of one surgical technique over another is best observed through the rate of complications and recurrences that result from the surgical procedure itself. Most published studies have confirmed the superiority of laparoscopy over open surgery.

Huang et al. compared open and laparoscopic technique in 233 infants with hypertrophic pyloric stenosis. They concluded that effectiveness of both the procedures did not differ between investigated groups but, similar to our findings, they also reported shorter operative time, shorter time to oral intake after surgery, and shorter length of hospitalization in the laparoscopic group of the patients [15].

Ismail et al. performed a prospective randomized clinical trial in 80 infants comparing clinical outcomes of open versus laparoscopic approach for the treatment of hypertrophic pyloric stenosis. They reported the superiority of the laparoscopic over open approach in regards to surgical times, hospital stay, and full postoperative feeding. These results are totally comparable to the results of our study. They also found lesser need of analgesia after surgery and better parents’ satisfaction in the laparoscopic group of the infants. They reported two cases of incomplete pyloromyotomy, as well as one wound infection in the laparoscopic group [29].

In contrast to previous reports, Zampieri et al. in their study in 60 infants did not find statistically significant difference in regards to outcomes of treatment and complication rates between two operative techniques and concluded that laparoscopic approach is a comparable alternative to the open approach [29]. Similar conclusion was drawn by Shawn et al. who reported, among 200 infants, only lesser pain after the procedure and fewer complication rates in the laparoscopic group [30]. Kim et al. compared laparoscopic pyloromyotomy with two open approach procedures: transverse incision below the right costal arch and circumbilical incision in 290 infants. They concluded that laparoscopic approach is safe and effective with the shortest operative time compared to open techniques [31].

Costanzo et al. investigated complications of procedure and duration of hospital stay between the open and laparoscopic approaches on a large cohort of 3256 infants with hypertrophic pyloric stenosis. They reported a significant decrease in overall morbidity in infants who received laparoscopic approach and concluded that in regards to postoperative outcomes laparoscopy may be superior over open surgery [17].

Shorter duration of surgery and length of hospital stay in laparoscopic approach has also been confirmed in the study of Mahida et al. among 1143 infants with hypertrophic pyloric stenosis [32].

Although the majority of published studies reported benefits of laparoscopic approach in the treatment of hypertrophic pyloric stenosis recent systematic reviews and meta-analyses, probably due to small sample sizes and relatively low incidence of disease, did not found significant evidence to support laparoscopic procedure over the open approach. Laparoscopy was generally associated with shorter operative times and length of hospital stay but slightly higher incidence of mucosal perforation and incomplete pyloromiotomies. No strong statistical significance for any of the above-mentioned variables was found or evidence was of very low-certainty [16,18,33,34,35].

Most of the authors for laparoscopic pyloromyotomy use a pyloric cutting knife. Pyloric cutting knife has been reported as safe but accidental injury to the surrounding organs or structures may occur and further complicate the procedure. Jain et al. in their study on 27 infants introduced the use of electrocautery hook for incision of the pylorus. They compared infants in whom pyloromyotomy was performed using a pyloric cutting knife with infants who received laparoscopic pyloromyotomy using electrocautery hook. They found both methods as safe and effective without complications [22]. The same results were recorded in our study. One of the main advantages of this technique is bloodless operative filed, which is very important to pediatric laparoscopic surgeons who work in very small spaces in infants and children [22]. Pediatric surgeon should always be careful because every surgical technique, in addition to having advantages, also has disadvantages. The main possible disadvantage is thermal damage or opening of the pyloric mucosa, if the electrocautery hook is inserted too deep, or lateral thermal damage to surrounding organs [22,36]. Care should be taken to avoid thermal damage as it does not have to be seen immediately, and delayed complications develop [22,27,36,37,38,39,40].

The retrospective character of the study as well as the limited number of the patients because of relatively low incidence of the disease is the most important limitation of the present study. Moreover, it was not possible to compare the treatment outcomes of electrocautery hook over pyloric cutting knife among our patients, because all the patients received laparoscopic pyloromyotomy using 3-mm electrocautery hook. Multicenter randomized studies on larger cohorts of the patients should be performed to confirm our findings and determine whether changing these parameters affect the outcomes of the study.

## 5. Conclusions

Both techniques are safe and effective for the treatment of infants with hypertrophic pyloric stenosis. Laparoscopic pyloromyotomy has benefits in shorter surgical time, lower frequency of postoperative vomiting, shorter length of hospital stay, and shorter time to oral intake after surgical procedure. Frequency of complications and rate of reoperations was also lower in laparoscopic approach but the difference was not statistically significant.

## Figures and Tables

**Table 1:** Demographic characteristics, laboratory data, and ultrasound findings of patients.

	Group I Open Pyloromyotomy (*n* = 61)	Group II Laparoscopic Pyloromyotomy (*n* = 64)	*p*
Demographic characteristics of patients			
Age (days)	34	31	0.453 *
median (IQR)	(23.5, 46)	(24, 38)	
Gender, *n* (%)			0.570 ^†^
Male	50 (82)	54 (85.7)	
Female	11 (18)	9 (14.3)	
Weight (g)	3 700	3 800	0.226 *
median (IQR)	(3525, 4240)	(3030, 4025)	
Malnutrition, *n* (%)	16 (26.2)	11 (17.8)	0.255 ^†^
Associated anomalies, *n* (%)	5 (8.9)	6 (10.3)	1.000 ^‡^
Laboratory data of patients			
Hypokalemia, *n* (%)	13 (21.6)	18 (29)	0.350 ^†^
Hypochloremia, *n* (%)	24 (39.3)	24 (37.5)	0.316 ^†^
Metabolic alkalosis, *n* (%)	54 (88.5)	55 (85.9)	0.974 ^†^
Ultrasound findings of patients			
Ultrasound—wall thickness (mm) median (IQR)	5.5 (5, 5.8)	5.2 (4.8, 6)	0.260 *
Ultrasound—length of pyloric canal (mm); median (IQR)	19 (18, 21.5)	19 (18, 21)	0.857 *
Ultrasound—diameter of pylorus (mm); median (IQR)	17 (15, 18)	18 (16, 18.5)	0.441 *

* Mann–Whitney U-test-test; ^†^ Chi-square test; ^‡^ Fisher’s exact test; IQR—interquartile range; n- number.

**Table 2:** Outcomes of treatment.

	Group I Open Pyloromyotomy	Group II Laparoscopic Pyloromyotomy	*p*
	(*n* = 61)	(*n* = 64)	
Surgical time (min)	45	35	0.00008 *
median (IQR)	(40, 57.5)	(30, 45)	
Time to oral intake (min)	22	6	<0.00001 *
median (IQR)	(13.5, 24)	(4, 8)	
Postoperative vomiting, *n* (%)	19 (31.1)	10 (15.6)	0.039 ^†^
Length of hospital stay (days)	6	3	<0.00001 *
median (IQR)	(4.5, 8)	(2, 3)	
Complications, *n* (%)	6 (9.8)	2 (3.1)	0.157 ^‡^
Perforation of mucosa	3 (4.9)	2 (3.1)	
Wound infection	3 (4.9)	0 (0)	
Reoperations, *n* (%)	3 (4.9)	0 (0)	0.113 ^‡^

* Mann–Whitney U-test-test; ^†^ Chi-square test; ^‡^ Fisher’s exact test; IQR—interquartile range.

## Data Availability

The data presented in this study is available upon request of the respective author. Due to the protection of personal data, the data is not publicly available.
